# A FIRST LOOK AT THE HEALTHY AGING INITIATIVE: YEAR ONE RESULTS

**DOI:** 10.1093/geroni/igae098.2884

**Published:** 2024-12-31

**Authors:** Talia Gilfix, Molly Quigley, Heather Andrews, Davide Cappon, Maggie Syme, Alvaro Pascual-Leone

**Affiliations:** Hebrew SeniorLife, Boston, Massachusetts, United States; Hebrew SeniorLife, Boston, Massachusetts, United States; Hebrew SeniorLife, Boston, Massachusetts, United States; Hebrew SeniorLife, Boston, Massachusetts, United States; Hebrew SeniorLife, Boston, Massachusetts, United States; Hebrew SeniorLife, Boston, Massachusetts, United States

## Abstract

The Healthy Aging Initiative (HAI) is a Boston-based longitudinal observational study assessing functional ability and well-being in 55+ adults residing in independent-living senior communities. Participants undergo assessments to evaluate modifiable risk factors for disability and dementia: mobility, cognition, strength, functional status, lifestyle factors, and mental wellness. Timely feedback/results are provided to empower meaningful changes. To gain insight into factors impacting functional ability, all participants’ data through Year 1 (N=173) were aggregated and key modifiable risk factors analyzed, revealing compelling trends across age groups and sex. Trends included sex differences among participants aged 80-84 years in the memory domain (Males 29% impaired (n=14); Females 13% impaired (n=24)). However, in the 85-89 age group these differences were no longer observed (Males 42% impaired (n=12); Females 39% impaired (n=23)), suggesting older women may “catch up” in impairment by their late 80’s. In mobility, females with high fall risk nearly doubled from age 75-79 to 80-84 (33% (n=18) to 63% (n=30)), while males in these age groups had the opposite effect (56% (n=9) to 27% (n=22)), indicating males who remain agile until the age of 80 may delay mobility decline. In lifestyle factors, it’s important to look at a domain from multiple perspectives. For instance, 68% of participants (n=132) report following recommended exercise guidelines, while 52% also report spending at least 6 hours sitting daily (n=101). Full results will be presented. As HAI expands and participants repeat assessments annually, critical insights may be obtained, creating a blueprint for translational clinical research in healthy ageing.

